# Citrullination in Rheumatoid Arthritis—A Process Promoted by Neutrophil Lysis?

**DOI:** 10.5041/RMMJ.10254

**Published:** 2016-10-31

**Authors:** Tal Gazitt, Christian Lood, Keith B. Elkon

**Affiliations:** Division of Rheumatology, University of Washington, Seattle, WA, USA

**Keywords:** Citrullination, complement activation, neutrophils, rheumatoid arthritis

## Abstract

Anti-citrullinated protein antibodies (ACPAs) are highly specific serologic markers for rheumatoid arthritis (RA) and can pre-date clinical disease onset by up to 10 years, also predicting erosive disease. The process of citrullination, the post-translational conversion of arginine to citrulline residues, is mediated by peptidylarginine deiminase (PAD) enzymes present in polymorphonuclear cells (PMNs). Calcium ions (Ca^2+^) are required for PAD activation, but the intracellular Ca^2+^ concentration in normal cells is much lower than the optimal Ca^2+^ concentration needed for PAD activation. For this reason, it has been proposed that PAD activation, and thus citrullination, occurs only during PMN cell death when PAD enzymes leak out of the cells into the extracellular matrix, or extracellular Ca^2+^ enters the cells, with the high Ca^2+^ concentration activating PAD. Recently, using artificial *in vitro* systems to corroborate their hypothesis, Romero et al. demonstrated that “hypercitrullination,” citrullination of multiple intracellular proteins, occurs within synovial fluid (SF) cells of RA patients, and that only modes of death leading to membranolysis such as perforin-granzyme pathway or complement membrane attack complex activation cause hypercitrullination. In order for Romero’s hypothesis to hold, it is reasonable to surmise that PMN-directed lysis should occur in the rheumatoid joint or the circulation of RA patients. Research conducted thus far has shown that immunoglobulin G (IgG) targeting PMNs are present in RA SF and mediate PMN activation. However, the role of anti-PMN IgG in mediating complement activation and subsequent PMN lysis and hypercitrullination has not been fully evaluated.

## CITRULLINATION IN RHEUMATOID ARTHRITIS

Citrullination, the post-translational conversion of arginine to citrulline residues by peptidylarginine deiminase enzymes (PADs), is thought to be an essential contributor to the rheumatoid arthritis (RA) disease pathogenesis.^1^ The potential role of citrullination in RA pathogenesis arises from several observations: rheumatoid arthritis patients have an increase in citrullinated proteins and PAD activity in mucosal surfaces as well as in synovial fluid;^2^,^3^ the citrullination reaction relevant to RA is mediated by two isoforms, PAD 2 and 4, both of which are present in neutrophils^4^,^5^ and which are the dominant PADs found in the synovial fluid of RA patients;^3^ and therapies that reduce PAD activity (including Cl-amidine, glucocorticoids, and paclitaxel) are associated with disease amelioration in animal models of arthritis.^6^–^8^ However, as none of the mentioned drugs selectively targets PADs, and Cl-amidine and its analogues as well as paclitaxel are yet to be tested in human RA, the therapeutic effect of PAD inhibition in human RA is still uncertain.

## ANTI-CITRULLINATED PROTEIN ANTIBODIES IN RA

Protein citrullination occurs not only in solution but also on cell-bound targets, including synovial fluid platelet-derived microparticles.^9^ Those citrullinated proteins, either cell-bound or in solution, may be targeted by autoantibodies to induce immune complex formation and subsequent FcγR-mediated inflammatory responses.^9^,^10^ The presence of autoantibodies directed against citrullinated proteins (anti-citrullinated protein antibodies, ACPAs) is considered a specific serologic marker for RA. Anti-citrullinated protein antibodies can often pre-date clinical disease onset by up to 10 years, also predicting erosive disease.^11^ Research involving preclinical RA cohorts points to a maturation process of the ACPA response with an increase in the number of autoantibodies to different citrullinated targets close to the onset of clinical disease, likely via epitope spreading.^12^,^13^ Further, evidence of ongoing B cell activation in response to citrullinated proteins in RA comes from the work of Tan et al.^14^ on the ACPA repertoire of RA patients showing differential epitope targeting by individual peripheral blood plasmablasts to citrullinated enolase, fibrinogen, and histone H2B. The discovery of well-developed IgA ACPA responses in at-risk individuals and in the stored serum of RA patients obtained at the preclinical phase of disease suggests that the immune pathogenesis occurs at mucosal surfaces in the earliest phases of RA and may serve as a trigger of systemic autoimmunity.^15^,^16^ Of particular relevance to this hypothesis is the finding that *Porphyromonas gingivalis* (a bacterium associated with periodontal disease,^17^ a condition which is more prevalent in new-onset, treatment-naive RA patients than healthy controls^18^,^19^) is uniquely able to modify arginine residues to citrulline by virtue of having its own isoform of PAD. This isoform is thought to citrullinate molecular targets not normally accessible to endogenous human PADs.^20^ Additional support for the mucosal origin of autoimmunity is work implicating the lung in RA disease pathogenesis. Several studies have reported that smoking, the greatest known environmental risk factor for RA development, increases the citrullination of lung proteins and is associated with ACPA formation specifically in patients with the HLA-DRB1*04:01 and *04:04 RA susceptibility haplotype.^11^,^21^

## CITRULLINATION AND THE RA SHARED EPITOPE

While it has long been appreciated that the close association between the shared epitope conferring RA susceptibility is tied to the expression of ACPAs,^22^ recent work has shed light on this association. Work by Snir et al.^23^ revealed that peptides derived from citrullinated vimentin bound more avidly to the binding pocket of HLA-DRB1*04:01 compared to the unmodified protein. This, in turn, leads to activation of CD4+ T cells reactive to citrullinated vimentin. Subsequent work by Scally et al.*,*^24^ aimed at elucidating the crystal structure of the HLA-DRB1*04:01/04 molecule, showed that citrulline was better accommodated within the electropositive binding pocket of HLA-DRB1*04:01/04 than the non-modified arginine. In contrast, the non-modified arginine was better accommodated by the electronegative binding pocket of the RA-resistant HLA DRB1*04:02 allomorph. Interestingly, the shared epitope also seems to function as an immune-stimulatory ligand that polarizes T cell differentiation towards type 17 T helper (Th17) cells, which are associated with autoimmunity.^23^

## NEUTROPHIL CELL DEATH AS A POTENTIAL CONTRIBUTOR TO CITRULLINATION IN RA

Despite this growing body of evidence supporting a role for citrullination in RA disease pathogenesis, an enigma regarding citrullination still remains: it is known that high calcium ion concentrations are required for PAD activation, but the intracellular calcium concentration in normal cells (10^−7^ mol/L) is much lower than the optimal calcium concentration needed for PAD activation (approximately 10^−5^mol/L).^4^ In an attempt to reconcile this discrepancy, PAD activation was proposed to be linked with neutrophil cell death, such that PADs leak out of the dead cells into the extracellular matrix, where the extracellular calcium concentration is sufficient for PAD activation (10^−3^mol/L). However, it was also considered that there could be influx of calcium ions from the extracellular space into the dying cells, promoting intracellular PAD activation.^4^ The precise form of cell death in relation to citrullination in RA has only recently been addressed.

NETosis, a specialized form of programmed necrosis of neutrophils in which the dying neutrophil extrudes an extracellular fibril matrix composed of chromatin fibers decorated with azurophilic granule-derived antimicrobial peptides and enzymes, is part of an important innate immune strategy to immobilize and kill invading micro-organisms.^25^ NETosis was suggested to contribute to ACPA formation in RA due to the citrullination of histone H3.^26^ Released neutrophil extracellular traps (NETs) not only expose citrullinated histone H3 as a potential antigen but also augment inflammatory responses in fibroblast-like synoviocytes which invade cartilage in RA.^27^ Indeed, recent research has shown that neutrophil death via NETosis plays an important role in RA pathogenesis. Enhanced NETosis in circulating and synovial fluid RA neutrophils may be impacted by inflammatory cytokines such as IL-17 and TNF-α as well as the presence of ACPAs.^1^,^28^ Research by Sohn et al.^10^ demonstrated that immune complexes (ICs) containing NET-derived citrullinated H2B from the RA synovium both activate macrophage TNF-α production and propagate NETosis *in vitro*, as well as induce ACPA-associated arthritis in a mouse model *in vivo*.

The notion of NETosis being sufficient to explain the broad range of citrullinated autoantigens to which ACPAs bind in RA has been questioned. In an attempt to explore the range of citrullinated targets and also the mode of cell death relevant to citrullination, Romero et al.^29^ examined a process that they called “hypercitrullination.” Hypercitrullination refers to citrullination of multiple intracellular proteins, which the authors showed does occur in cells obtained from RA synovial fluid. Using *in vitro* reagents, Romero et al.^29^ revealed that hypercitrullination depended on two specific immune-mediated membranolytic pathways: one mediated by cytotoxic cells through the perforin-granzyme pathway, and the other mediated by complement activation and formation of the membrane attack complex. Their findings were corroborated in a recent publication by Zhou et al.,^30^ who demonstrated that membranolytic agents that trigger a sufficient influx of extracellular Ca^2+^ induce a marked citrullination of multiple proteins in human neutrophils and monocytes and, to a lesser extent, in T lymphocytes and natural killer cells.

In order for Romero’s hypothesis to hold, it is reasonable to surmise that neutrophil-directed lysis should occur in the rheumatoid joint or the circulation of RA patients. Complement activation, a potential mediator of membranolytic cell death, has been shown to occur in the RA synovium^31^ and has long been known to participate in recruitment and activation of neutrophils in RA.^32^ Research conducted by Starkebaum and colleagues at the University of Washington^33^–^36^ revealed that ICs, in particular IgG targeting neutrophils, are present in RA synovial fluid (SF) and mediate neutrophil activation. Although the literature contains conflicting data regarding the nature and prevalence of anti-neutrophil-binding IgG in RA depending on the method used for analysis of antigenic targets,^33^,^34^,^37^,^38^ it is estimated that IgG-containing ICs as well as complement C3 activation fragments can be found in the joints of >90% of RA patients.^39^,^40^

## COMPLEMENT ACTIVATION AS A POTENTIAL CONTRIBUTOR TO HYPERCITRULLINATION IN RA

Because IgG is known to activate complement, neutrophil-directed IgG likely participates in neutrophil lysis by complement activation. In keeping with the hypothesis of IgG-mediated complement activation in SF, Bedwell et al.^32^ and Swaak et al.^41^ both showed that high levels of aggregated IgG bearing the C3 complement activation breakdown component C3d can be found in SF of RA patients, correlating with level of complement. Further, Brodeur et al.^42^ showed that SF levels of complement C5b-9 and fragment Bb are elevated in patients with RA. The limitation of these findings, however, is that they do not conclusively support complement activation in the joint, as the source and/or target of complement activation, e.g. the underlying mechanism(s) driving complement activation, cannot be addressed by assessing complement split products in solution. Complicating matters further is the difficulty distinguishing ICs from anti-neutrophil-binding antibodies on the cell surface of neutrophils, which has made it extremely difficult to determine the precise antigenic specificity of anti-neutrophil-binding IgG in RA. Interestingly, anti-neutrophil-binding IgG has been shown to play an important role in mediating neutropenia in Felty’s syndrome,^33^,^34^,^43^ an RA-associated disease in which severe arthritis and neutropenia prevail. However, precisely how anti-neutrophil-binding IgG leads to neutropenia in these patients, and whether this plays a role in complement-mediated neutrophil lysis and hypercitrullination in RA in general, remains to be clarified.

## CD8+ T CELL MEDIATED, PMN-DIRECTED CYTOTOXICITY AS A POTENTIAL CONTRIBUTOR TO HYPERCITRULLINATION IN RA

Historically, the role of CD8+ T cells in RA has been overshadowed by the unequivocal role of DRB1 MHC II susceptibility, and hence CD4+ T cells, in the disease. However, it is well known that CD4+ T cells prime CD8+ T cells for effector function.^44^ Recently, de Hair and colleagues^45^ emphasized the importance of CD8+ T cells in early RA through detection of perforin and granzyme-producing CD8+ T cells in the synovium of patients with preclinical RA. Carvalheiro et al.^46^ similarly demonstrated abundant perforin/granzyme+ CD8+ T effector cells in the peripheral blood and SF from active RA patients. They showed that this effector T cell phenotype persisted in disease remission, thus maintaining the potential for disease relapse. Remarkably, in the K/BxN chronic mouse model of RA, depletion of CD8+T cells by thymectomy (to prevent new cells from emerging) cured the mice of RA.^47^ Together, these studies support the hypothesis that CD8+ T cell-mediated cytotoxicity (cytotoxic T lymphocytes) may indeed play an important role in promoting synovial inflammation and immune propagation in RA patients. Because polymorphonuclears are the most abundant cell type found in inflammatory SF in the RA joint, these cells may be a target of cytotoxic cells; however, this hypothesis has yet to be explored.

## CONCLUSION

In summary, while evidence is accumulating in support of citrullination of multiple proteins as a potential driver in RA disease pathogenesis, direct proof linking membranolytic pathways as contributors to hypercitrullination in RA patients beyond artificial *in vitro* systems is still lacking. Moreover, future research in the field should establish whether there is a causative relationship between complement activation and neutrophil lysis with neutrophil-directed cytotoxicity occurring preferentially in the RA joint and promoting hypercitrullination.

## Abbreviations

ACPAs: anti-citrullinated protein antibodies
ICs: immune complexes
NETs: neutrophil extracellular traps
RA: rheumatoid arthritis
PADs: peptidylarginine deiminase enzymes
SF: synovial fluid
